# Assessment of Client's Perception in Terms of Satisfaction and Service Utilization in the Central Government Health Scheme Dispensary at Kolkata

**DOI:** 10.4103/0970-0218.40883

**Published:** 2008-04

**Authors:** D Haldar, AP Sarkar, S Bisoi, P Mondal

**Affiliations:** Department of Community Medicine, BMC, Burdwan, India; 1 Department of Community Medicine, IPGMER, Kolkata, India; 2 Department of Community Medicine, CMC, Kolkata, India

## Introduction

All healthcare providers and programmes in our country have overwhelming emphasis on quantitative aspect of service delivered, which means that, in a quest to chase runaway targets, we neglect the concept of quality of care, which is also a right of clients.(1) Healthcare providers and programmes worldwide have increasingly recognized that the quality of care they provide determines their overall success in attracting the clients and meeting their needs, and the quality improvement initiative has been started because poor quality is costly - to clients, to programmes and to the society overall. People's perception about quality of care often determines whether they seek and continue to use services.(2) Being intangible in nature, the perception directly affects the quality rating in service. So, there are attempts to scale and measure this perception. OPD is the window to any health system and OPD care indicates the quality care of hospital reflected by patient's perception in terms of satisfaction to the services they are provided. Scarcity of information on this aspect of health care inspired the authors to carry out the present study at the Central Government Health Scheme (CGHS) Dispensaries, Kolkata, with a view to (1) assess the client's satisfaction about structures and processes running in CGHS and (2) know desired level of services as perceived by the patients.

## Materials and Methods

It was an institution-based cross-sectional study, conducted *from August'02 to Jan'03* at OPD facility of CGHS dispensaries at Kolkata, serving Central Government Employees, Pensioners, Freedom fighters, Ministers, MP's, Judges along with their dependents, through network of Allopathic, Homeopathic and Ayurvedic dispensaries which procured drugs purchased mainly from GMSD (Govt. Medical Store Depot.). Allopathic system provides OPD service, emergency care, laboratory investigations, Maternal & Child Health (MCH), Family Planning service (FP), referral for specialist/indoors care in approved hospitals, drug supply etc. Multistage sampling technique was adopted starting with selection of four allopathic dispensaries, namely, Dover lane, Lake, Regent estate and Mint colony out of the existing 17 dispensaries at Kolkata, by simple random sampling method using random number. In the second stage, two Focus Group Discussion (FGD) were held at each dispensary, one at an early hour and the other at late hour, and FGD being a one-time event with all participants, 12-15 respondents for each FGD were selected at a time from OPD queue, existing at that moment, by simple random sampling technique. Finally, 30 patients from Dover lane, Lake, Mint colony each and 32 patients from Regent estate dispensaries; thus, a total of 122 patients were selected from OPD queue via systematic random sampling technique. Simple proportion was used for data analysis. Obtaining written permission, data were collected prospectively by interviewing those 122 patients (selected at the third stage) with semi-structured questionnaire developed on the basis of salient opinions gathered from eight FGDs held at four dispensaries (two at each), focusing on different issues of CGHS; containing few questions for assessment of client's perception about structure, various service processes and few to assess the outcome of CGHS health care plus level of service desired by the users.

## Results and Discussion

Analysis revealed that only 22.9% participants could avail CGHS dispensary service during emergency health problem, and short service hours (56.8%) plus distance from residence (55%) were reported as predominant inconvenience for availing service. Dr. Shah Hossain, in his study, found 74% dissatisfied respondents to unsuitable dispensary timings.(3) Similar observations were made by Nandan in rural area of Agra and Elshabrawy *et al.* in rural area of Riyadh (Saudi Arabia).(4,5) Overall, 60.6% and 65.5% responses went in favour of general facility and doctor's advice, respectively, as the best services. 39.3%, 23.0% and 19.7% respondents indicated indoor treatment system in non-CGHS hospitals, drug supply and long queue, respectively, as the worst issues; of course, 23.0% failed to identify anything worst in their dispensaries. However, Hossain found that 73% clients experienced either long queue or uncomfortable waiting hall arrangement, and 71% clients thought that the drugs prescribed were either not available at dispensaries or were of poor standard.(3) Elshabrawy *et al.* also found long waiting time as a cause of dissatisfaction.(5) Overall, 50.8% participants preferred to dispensary's own laboratory and 31.1% to a perceived reliable laboratory anywhere at Kolkata as their best choice, contrary to Dr. Hossain's observation where 80% respondents ranked Pvt. laboratory as superior than that of CGHS. Banerjee in her study in a central government urban health center, Kolkata, found laboratory investigations were to be the second most utilized service (70.50%).(6) Respondent's suggestions for betterment of CGHS were noted and found that 78.7% respondents suggested for the improvement in the system of drug supply and 67.2% also suggested for increased service hours i.e. 24 hours service [Table 1]. Similar observations were also made by other investigators.(4) According to Dr. Hossain, 71% clients believed that CGHS health service could be improved by enhancing drug supply. Participants expressed their concern for attitude and sincerity of healthcare providers of approved hospitals for indoor treatment plus specialists care (Nandan in his study in Lalitpur and Jhansi and Misra from a study in Mahoba found that dissatisfaction among community for Primary Health Center (PHC) were due to lack of caring and sympathetic behaviour of doctors and non-availability of drugs) and 77%, 80.3% and 55.7% respondents expressed their needs for CGHS's own indoor facility, specialists in all disciplines and ambulance facility at dispensary level, respectively [Table 2]. Elshabrawy *et al.* found 38.9% dissatisfied patient due to the absence of specialist clinics.(5) A comparison was made, considering cost, appropriateness and quality of service offered by CGHS and other health system at Kolkata; 59.0% respondents ranked CGHS as better than other health systems (govt. and pvt.), of course 37.7% participants failed to rank their dispensaries. This was contrary to the finding of NFHS-II and other studies (MIRA study of Panwar), where pvt. sector facilities were rated higher than that of public sector. But Banerjee showed that nearly 2/3^rd^ (61.5%) respondents expressed their satisfaction by ranking MCH services of UHC, Kolkata, as excellent (28.5%) and good (33.0%).(6) Lastly, more than 2/3^rd^ (68.8%) participants mentioned that they availed dispensary service regularly, i.e. for all times for all health problems (other than emergency, in some cases). Banerjee also found 62.75% respondents to avail all services from the urban health center, Kolkata.(6)

**Table 1:** Frequency distribution of respondents according to their suggestions for the improvement of CGHS services *

Category of options	Dover lane *n* = 30; No., (%)	Lake *n* = 30; No., (%)	Regent estate *n* = 32; No., (%)	Mint colony *n* = 30; No., (%)	Total *N* = 122; No., (%)
Improvement of drug supply	24 (80.0)	14 (46.6)	28 (87.5)	30 (100)	96 (78.7)
To increase the number of doctors and other staffs	2 (6.6)	12 (40.0)	10(31.3)	18 (60.0)	42 (34.4)
HCPs need regular training to improve the quality of services†	12(40.0)	16(53.3)	8(25.0)	4 (13.0)	40 (32.8)
To increase the number of dispensary	14 (46.6)	10(33.3)	6(18.8)	14 (46.6)	44 (36.0)
To increase the service hours	12(40.0)	28 (93.3)	32 (100.0)	10 (33.3)	82 (67.2)

* Multiple responses

† HCP = Healthcare provider

**Table 2:** Frequency distribution of the responses according to the felt-need of the respondents

Felt needs of the respondents	Dover lane *n* = 30; No., (%)	Lake *n* = 30; No., (%)	Regent estate *n* = 32; No., (%)	Mint colony *n* = 30; No., (%)	Total *N* = 122; No., (%)
Ambulance facility	20 (66.6)	14 (46.6)	24 (75)	10(33.3)	68 (55.7)
Immunization	4(13.3)	6 (20.0)	10(31.3)	12 (40.0)	32 (26.2)
Own indoor hospital	22 (73.3)	22 (73.3)	24 (75)	26 (86.6)	94 (77.0)
Specialists in all disciplines	26 (86.6)	26 (86.6)	22 (68.8)	24 (80.0)	98 (80.3)
Homeopath/Ayurved care in their dispensary	6 (20.0)	16(53.3)	8(25)	16(53.3)	26 (37.7)

## Conclusion

Client's satisfaction was reflected by their happy expression about general facility, doctor's consultations, superiority of their health system and also by consistent regular utilization by majority. On the contrary, they were dissatisfied with unsuitable/inadequate service hours, long waiting time, indoor treatment system, drug supply and non-existence of CGHS's own laboratory. Their felt-need for long service hours, improved drug supply, own indoor facilities and specialists in all disciplines and ambulance facility at dispensary level was to be addressed as priority to secure better participation for ultimate success of CGHS.
